# Optimization of membrane dispersion ethanol precipitation process with a set of temperature control improved equipment

**DOI:** 10.1038/s41598-020-75900-1

**Published:** 2020-11-04

**Authors:** Jingjing Pan, Yanni Tai, Haibin Qu, Xingchu Gong

**Affiliations:** Pharmaceutical Informatics Institute, College of Pharmaceutical Sciences, Zhejiang University, Hangzhou, 310058 China

**Keywords:** Chemical engineering, Green chemistry, Drug development

## Abstract

Ethanol precipitation is an important separation and purification process in the traditional Chinese medicines (TCMs) industry. In the present study, a membrane dispersion micromixer was applied to achieve good mixing for the ethanol precipitation process of *Astragali radix* concentrate. New experimental apparatus was set up to rapidly lower the temperature of ethanol solution before mixing with the concentrate. Ethanol precipitation process was optimized according to Quality by design concept. To identify critical material attributes (CMAs), ten batches of *Astragali radix* were used to prepare concentrates. Calycosin-7-O-β-D-glucoside content, the sucrose content, and the electrical conductivity were found to be CMAs after the correlation analysis and stepwise regression modelling. Definitive screening design was used to investigate the relationships among critical process parameters, CMAs, and process critical quality attributes (CQAs). Quadratic models were developed and design space was calculated according to the probability of attaining process CQA standards. A material quality control strategy was proposed. High quality and low quality *Astragali radix* concentrates can be discriminated by the inequalities. Low quality *Astragali radix* concentrates should not be released for ethanol precipitation process directly. Verification experiment results indicated accurate models and reliable design space. The temperature control method and control strategy are promising for ethanol precipitation process of other TCMs or foods.

## Introduction

Ethanol precipitation is a widely applied separation technology in the production of traditional Chinese medicines (TCMs)^1^. Ethanol precipitation has many advantages, including low cost, convenient operation, and safe solvent^2–4^. Polar impurities, such as proteins and sugars, can be at least partly removed in ethanol precipitation^5–9^. However, ethanol precipitation process suffers from loss of active ingredients, low batch-to-batch consistency of supernatant composition, and long standing time^10–12^. These problems are mainly caused by low efficient equipment and imperfect control strategy.

In industry, ethanol precipitation process is usually performed in a stirring tank by adding an ethanol solution to a TCMs concentrate^3^. However, large density difference between ethanol solution and concentrate, high viscosity of concentrate, and rapid formation of precipitate all result in poor mixing of the two phases^13,14^. Because precipitate easily encapsulates some concentrate, the encapsulation loss of active ingredient is commonly observed in industry. After adding ethanol solution, a long standing time is usually required to let the active ingredients encapsulated dissolve to ethanol phase slowly. The low heat exchange efficiency of the stirring tank is another reason for long standing time because it is time consuming to cool the precipitation system. Recently, the mixing of ethanol and concentrates was intensified by using a membrane dispersion micromixer, and the encapsulation loss of active components was effectively reduced^13^. At present, membrane dispersion micromixer was mainly used to prepare nanoparticles or perform other synthetic reactions^15–18^. The effects of temperature, phase ratio, and flow rate on reaction performance were studied^15–18^. However, there is little research on the temperature control of ethanol precipitation process.

According to the concept of Quality by design (QbD), operating a pharmaceutical process with the design space containing the acceptable ranges of material attributes and process parameters is an effective way to improve the batch-to-batch consistency^19–25^. There are some published works on the development of design space for ethanol precipitation process control^26–29^. However, only the concentrate attributes that are easily controlled in upstream concentration process were concerned, such as water content, dry matter content, and concentrate density^2,30^. These concentrate attributes are usually considered as controllable material attributes.

According to Yan et al. and Zhang et al., active ingredient content, and other physical or chemical properties may also be critical material attributes (CMAs) of an ethanol precipitation process^31,32^. These properties were mainly affected by medicinal material quality and change among different batches. These concentrate attributes are considered as uncontrollable material attributes in this work. In the published works^27,30^, ethanol precipitation design space was developed without the consideration of uncontrollable material attributes, which lead to unsatisfactory control of batch-to-batch consistency. Therefore, design space considering controllable and uncontrollable material attributes, critical process parameters (CPPs), and process critical quality attributes (CQAs) is required for the control of ethanol precipitation process.

**Figure 1:**
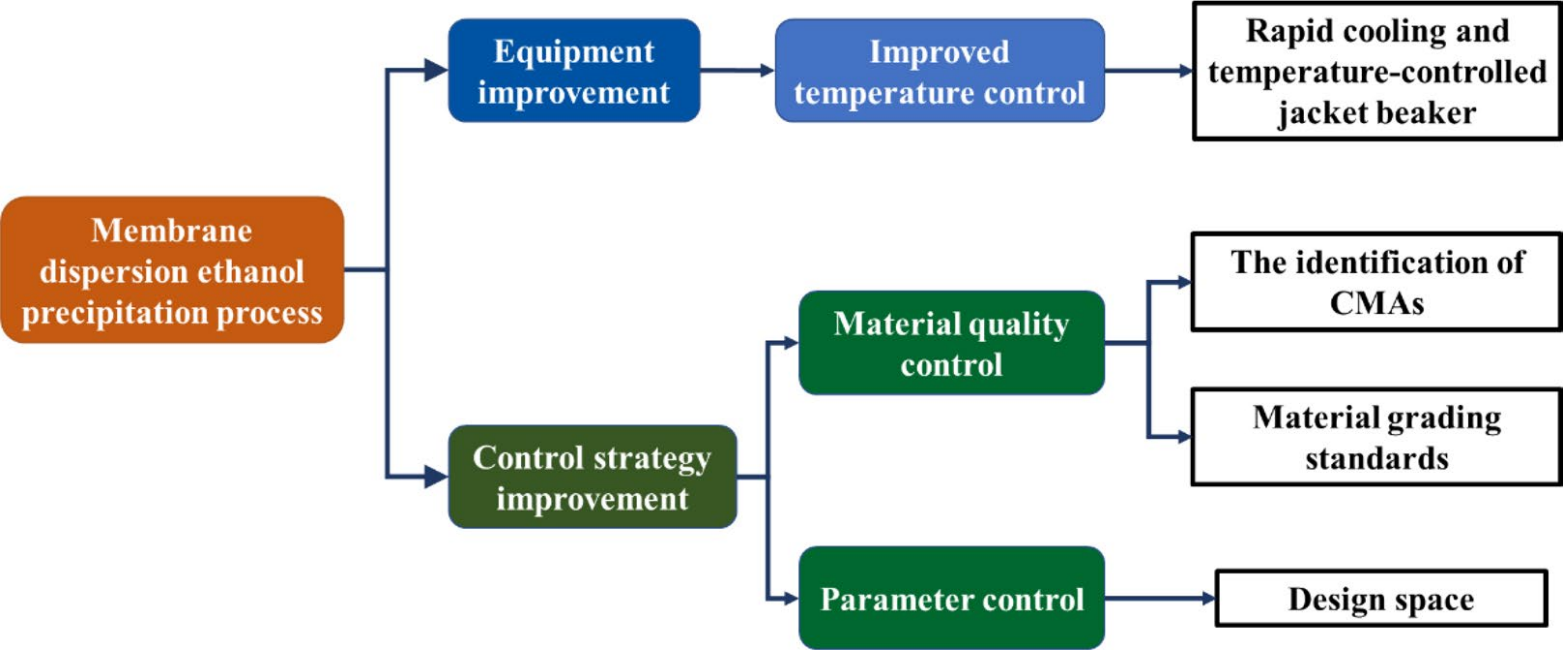
Schematic diagram of this work.

In this work, a membrane dispersion micromixer was used for continuously adding ethanol solution to *Astragali radix* concentrates. Temperature control was enhanced by rapid cooling. Ethanol precipitation process was optimized according to a design space approach. CMAs of *Astragali radix* extracts were identified. Definitive screening design was used to investigate the relationships among CPPs, CMAs and CQAs. The models were then developed and the design space was calculated. A material quality control strategy considering the requirements of manufacturing processes was proposed. Verification experiments were carried out for high quality *Astragali radix* concentrates. Figure 1 is a schematic diagram of this work.

## Experiment

### Materials and chemicals

Ten batches of *Astragali radix* were collected from Yunnan province and Gansu province of China. Reference substances of Astragaloside IV, Astragaloside II, Calycosin-7-O-β-D-glucoside (CG), 9,10-dimethoxypterocarpan-3-O-β-D-glucoside (PG), 2-hydroxy-3′,4′-dimethoxyisoflavane-7-O-β-D-glucoside (IFG) were purchased from Shanghai Winherb Medical Technology Co., Ltd. (Shanghai, China). The standard substances of D-fructose (99.5%) and sucrose (99%) were supplied by Aladdin Chemistry Co., Ltd. (Shanghai, China) and Sigma-Aldrich Co., Ltd. (Shanghai, China), respectively. Acetonitrile (HPLC grade) and methanol (HPLC grade) were purchased from Merck (Darmstadt, Germany). Formic acid (HPLC grade) was purchased from ROE scientific Inc. (Newark, America). Na_2_CO_3_ (analytical grade) and ethylene glycol (analytical grade) were obtained from Sinopharm Chemical Reagent Co., Ltd. (Shanghai, China). Triethylamine (HPLC grade) was purchased from Aladdin Chemistry Co., Ltd. (Shanghai, China). Ethanol (analytical grade) was purchased from Shanghai Lingfeng Chemical Reagent Co., Ltd. (Shanghai, China). Deionized water was prepared by an academic water purification system (Milli-Q, Milford, MA, USA).

### Preparation of *Astragali radix* concentrate

The *Astragali radix* was reflux-extracted with water of 6 mL/g decoction piece water twice. Extraction time of each extraction was 0.5 h. The two extracts were obtained by filtration and then combined. The combined extracts were concentrated under reduced pressure to obtain the concentrate. The temperature was controlled at about 70 °C and the pressure was about 50 mbar during concentration, and the concentrate density at the end point was about 1.2 g/mL.

### Apparatus and ethanol precipitation process

**Figure 2:**
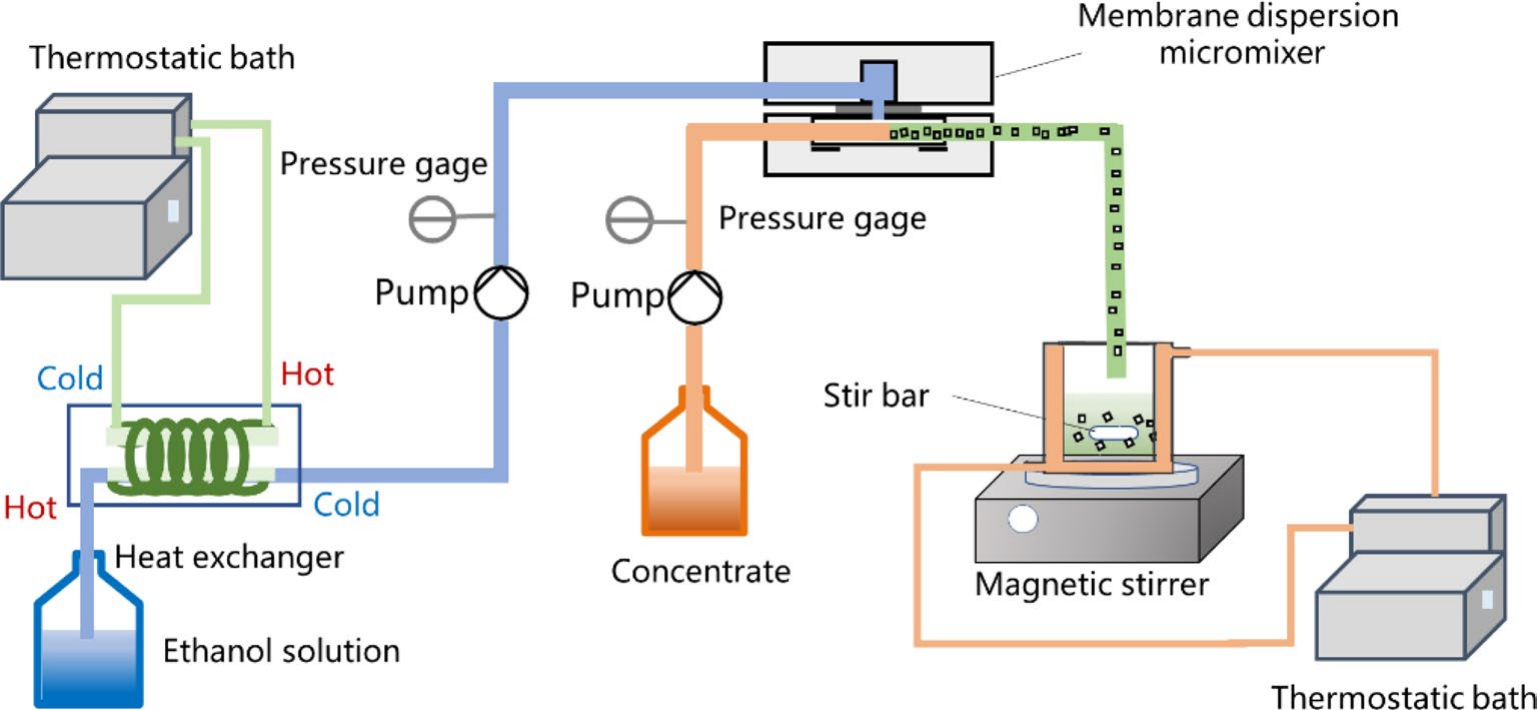
Schematic diagram of the experimental setup.

Figure 2 is the schematic diagram of the experimental setup. The membrane dispersion micromixer employed in this experiment was detailly described in previous work^13^. The sizes of mixing chamber (8 × 1 × 0.5 mm) were controlled by PTFE gaskets. The average pore size of stainless-steel membrane was 18 μm.

Ethanol solution at room temperature was cooled with a heat exchanger (K030-20M-NB4, Ningbo Gaori Technology Co., Ltd.). The heat exchanger was connected to the refrigeration circulation device (CA-1116A, Tokyo Rikakikai, Co. Ltd.). The heat transfer medium in the heat exchanger was 60% ethylene glycol–water (v/v). The low-temperature ethanol solution was served as the dispersed phase pumped into the micromixer by a gear pump (CT3001F, Baoding Reef Fluid Technology Co., Ltd.). A concentrate was pumped into the micromixer by an advection pump (2PB-20005II, Beijing Xingda Technology Development Co., Ltd.) as the continuous phase. The outlet mixture was collected in a jacketed beaker and magnetically stirred for 5 min. The temperature of the jacketed beaker was controlled by a thermostatic bath (THYD-1030W, Ningbo Tianheng instrument factory). The medium in the thermostatic bath connected to the jacketed beaker is 30% glycerol–water (v/v). The supernatant was collected after filtration. After an experiment, the apparatus was washed with 0.05% (wt.) Na_2_CO_3_ solution and ethanol sequentially.

The low-temperature ethanol solution and a temperature-controlled jacketed beaker were used to control the temperature of the mixture together. The use of low temperature ethanol solution could reduce the temperature of the mixture in a short time. The temperature controlled jacketed beaker was used to control the temperature precisely. The temperature ranges are shown in Table S1.

### Experimental design

#### The evaluation of ethanol precipitation process

The main active components of *Astragali radix* were flavonoids and saponins. In Chinese Pharmacopeia, CG and Astragaloside IV are chosen as the representatives for flavonoids and saponins, respectively. In this study, three flavonoids of CG, PG, IFG, and two saponins of Astragaloside IV and Astragaloside II were measured as the representative active components of *Astragali radix*. In this work, the purity of five active components and dry matter removal were selected as the process CQAs.

#### CMAs identification

To identify the CMAs, the experiments were carried out with different concentrates under fixed process conditions. The mass ratio of ethanol solution to concentrate (ECR) was 1.5 g/g, the dry matter content of concentrates was 45%, the ethanol solution concentration was 92% (v/v), the refrigeration temperature was 15 °C, and the flow rate of concentrate was 60 mL/min. The electrical conductivity, flavonoid contents, saponin contents, and sugar contents of different concentrates were measured.

#### Definitive screening design

After identifying the CMAs, the definitive screening design was used to study the quantitative relationships among the potential CPPs, CMAs, and process CQAs. Many parameters can be studied in a very small number of experiments with the definitive screening experiment design^33^. Dry matter content (X_1_), ECR (X_2_), the ethanol solution concentration (X_3_), and the temperature (X_4_) were selected as potential CPPs because they were found to be CPPs in published works^30,34^. The experimental conditions are listed in Table 1. To study the effects of materials, different concentrates of *Astragali radix* were used in these experiments, as shown in Table 1.

**Table 1:** Conditions and results of definitive screening designed experiments.

Experimental no	*Astragali radix* concentrate	Process parameters (coded variable)				Purity of flavonoids and saponins in the supernatant (μg/g dry matter)					Dry matter removal (Y_6_)
		Dry matter content (X_1_, %)	ECR (X_2_, g/g)	The ethanol solution concentration (X_3_, %)	Temperature (X_4_, °C)	Astragaloside IV (Y_1_)	Astragaloside II (Y_2_)	CG (Y_3_)	PG (Y_4_)	IFG (Y_5_)	
11	N1	40(− 1)	2.0(+ 1)	92(0)	25.0(+ 1)	1582	1544	1734	865.3	826.2	0.395
12	N2	40(− 1)	2.0(+ 1)	89(− 1)	5.0(− 1)	1488	1003	2217	1088	1120	0.459
13	N3	45(0)	1.5(0)	92(0)	15.0(0)	2596	2447	2199	1476	1356	0.384
14	N7	50(+ 1)	1.0(− 1)	95(+ 1)	25.0(+ 1)	692.4	763.6	1279	348.3	359.3	0.461
15	N10	40(− 1)	1.0(− 1)	95(+ 1)	15.0(0)	235.6	162.7	1534	332.2	208.5	0.331
16	N6	50(+ 1)	1.0(− 1)	92(0)	5.0(− 1)	244.9	346.7	1761	367.0	237.3	0.373
17	N2	40(− 1)	1.5(0)	95(+ 1)	5.0(− 1)	1831	1294	2643	1317	1316	0.448
18	N8	45(0)	2.0(+ 1)	95(+ 1)	25.0(+ 1)	496.7	527.2	1907	388.8	303.3	0.547
19	N9	50(+ 1)	1.5(0)	89(− 1)	25.0(+ 1)	368.7	758.3	2133	531.3	306.0	0.447
20	N5	50(+ 1)	2.0(+ 1)	95(+ 1)	5.0(− 1)	852.1	867.6	2350	479.9	486.6	0.561
21	N4	45(0)	1.0(− 1)	89(− 1)	5.0(− 1)	1465	1540	2317	1168	897.4	0.378
22	N8	40(− 1)	1.0(− 1)	89(− 1)	25.0(+ 1)	373.6	292.3	1223	195.5	327.4	0.285
23	N10	50(+ 1)	2.0(+ 1)	89(− 1)	15.0(0)	505.6	265.1	1613	258.6	430.2	0.385
24	N3	45(0)	1.5(0)	92(0)	15.0(0)	2548	2527	2205	1418	1214	0.365
25	N3	45(0)	1.5(0)	92(0)	15.0(0)	2409	2368	2112	1357	1172	0.356
26	N3	45(0)	1.5(0)	92(0)	15.0(0)	2554	2514	2171	1390	1197	0.371

### Analytical methods

The contents of Astragaloside IV, Astragaloside II, CG, PG, and IFG were determined using a HPLC-ELSD method developed by Luo et al.^35^. A HPLC (1260, Agilent Technologies, USA) system with a UV detector and ELSD detector was used. Samples were diluted with 50% (v/v) methanol solution. Analyses were conducted on a Zorbax SB-C18 column (4.6 mm × 250 mm, 5 μm) with the column temperature controlled at 30 °C. The flow rate of solvent was maintained at 0.8 mL/min, while the injection volume of sample was set at 10 μL. The detection wavelength was fixed at 270 nm. The ELSD operation parameters were as follows: the evaporator temperature was fixed at 30 °C, nebulizer temperature was fixed at 80 °C, and gas flow rate was fixed at 1.6 L/min. The mobile phase was consisted of solvent A (0.2% (v/v) formic acid in water) and solvent B (acetonitrile). The solvent gradients were as follows: 0–16 min, 15–23% B; 16–20 min, 23–28% B; 20–25 min, 28–30% B; 25–30 min, 30% B; 30–40 min, 30–55%B; 40–50 min, 55–95% B. The signals of CG, PG, and IFG were detected by the UV detector. The signals of Astragaloside IV and Astragaloside II were detected by the ELSD detector. A typical chromatogram of *Astragali radix* supernatant was shown in Figure S1.

The HPLC method developed by Shao et al. was used to determine the contents of d-fructose and sucrose^36^. The HPLC system (1260, Agilent Technologies, USA) was equipped with an Alltech 2000ES ELSD detector. All the samples were diluted with 85% (v/v) acetonitrile–water mixture and carried out on a XBridge Amide column (5 μm, 4.6 × 250 mm; Waters, Milford, MA, USA). The column temperature was fixed at 34 °C. The solvent flow rate was fixed at 0.9 mL/min and the sample injection volume was set at 5 μL. The mobile phase solvent A was 0.3% (v/v) triethylamine in water and solvent B was 0.3% (v/v) triethylamine in acetonitrile. The solvent gradients were as follows: 0–37 min, 85–76% B; 37–38 min, 76–60% B; 38–48 min, 60–100% B. The re-equilibrium time was 10 min. The ELSD operation parameters were as follows: the nebulizer temperature was set at 65 °C, evaporator temperature was set at 60 °C, and gas flow rate was set at 1.8 L/min. A typical chromatogram of *Astragali radix* concentrate was shown in Figure S2.

Dry matter content was determined using a gravimetric method as described in previous work^28^. Each sample of the concentrates was diluted with water to a solution of 2% dry matter content. The conductivity of the diluted concentrates was measured using a portable conductivity meter (DDBJ-350, Hangzhou Qiwei Instrument Co., Ltd.) at 25 °C. The viscosity of an *Astragali radix* concentrate was measured with a viscometer (NDJ-8SN, Shanghai Precision and Scientific Instrument Co., Ltd.)

### Data processing

The calculation formulas of active component purity and dry matter removal are as follows:

1 $$ Active \;component\;{\text{purity }} = \frac{{AC_{s} }}{{DM_{S} }} $$

2 $$ Dry\, matter\;removal = \left( {1 - \frac{{m_{s} \times DM_{s} }}{{m_{c} \times DM_{c} }}} \right) \times 100{\text{\% }} $$

where *m*, *AC*, and *DM* stand for mass, active component contents, and dry matter contents, respectively; subscripts *S* and *C* represent supernatants and concentrates, respectively.

To identify CMAs, Eq. (3) was used to model material attributes and process CQAs.

3 $$ Y = a_{0} + \mathop \sum \limits_{k = 1}^{8} c_{k} Z_{k} $$

where *Y* is the process CQAs; *a*_0_ is a constant; *Z*_*k*_ represents a material attribute; and *c*_*k*_ is the partial regression coefficient. Insignificant variables were removed by stepwise regression. The significance levels for adding terms and removing terms were both set to 0.1. The material attributes remaining in the model after stepwise regression were considered to be the CMAs.

Quadratic models were developed based on the definitive screening designed experiment results. Equation (4) was used to model CPPs, CMAs, and process CQAs.

4 $$ Y = a_{0} + \mathop \sum \limits_{i = 1}^{n} b_{i} X_{i} + \mathop \sum \limits_{i = 1}^{n} b_{ii} X_{i}^{2} + \mathop \sum \limits_{i = 1}^{n - 1} \mathop \sum \limits_{j = i + 1}^{n} b_{ij} X_{i} X_{j} + \mathop \sum \limits_{k = 1}^{m} d_{k} Z_{k}^{C} $$

where *n* and *m* are the number of CPPs and CMAs, respectively; *b* and *d* are the partial regression coefficients; *X*_*i*_ is a potential CPP; and *Z*^C^ is a CMA. Stepwise regression was performed as before decribed. Data analysis was performed by Design Expert (version 11.0.0, Stat-Ease Inc., USA).

## Results

### Material attributes

The quality attributes of different *Astragali radix* concentrates are shown in Table 2. The electrical conductivity was between 1442 and 2390 μS/cm, indicating different electrolyte contents in concentrates. The content of Astragaloside IV and Astragaloside II was lower than 2000 μg/g dry matter. The content of CG among the three flavonoid contents was the highest, which can exceed 1600 μg/g. The other flavonoids were less than 1000 μg/g dry matter. The sucrose content was higher than the d-fructose content, which could be more than 700 mg/g dry matter. At most occasions, sucrose was the main component of dry matter. The d-fructose content was lower than 30 mg/g dry matter.

**Table 2:** Quality attributes different batches of *Astragali radix* concentrates.

Concentrate number	Electrical conductivity (dry matter content of 2%) (Z_8_, μS/cm_)_	Contents of flavonoids and saponins (μg/g dry matter)					Sugar contents (dry matter)	
		Astragaloside IV (Z_1_)	Astragaloside II (Z_2_)	CG (Z_3_)	PG (Z_4_)	IFG (Z_5_)	d-fructose (Z_6_)	Sucrose (Z_7_)
N1	1944	1062	1200	1119	641.6	471.5	25.03	568.0
N2	1832	1020	763.4	1490	688.7	725.6	27.26	489.0
N3	2390	1887	1948	1550	1000	881.2	11.07	616.2
N4	1995	928.6	1093	1693	794.2	603.4	25.48	631.8
N5	1490	390.0	478.9	1121	272.9	228.6	18.54	714.4
N6	1667	247.1	314.3	1390	303.0	250.3	24.35	732.7
N7	1597	321.2	558.7	839.2	190.0	288.4	19.65	706.5
N8	1442	314.7	291.5	1067	207.4	179.2	20.02	695.6
N9	1655	284.5	562.1	1401	341.9	191.4	15.82	670.0
N10	1534	371.4	178.3	1445	299.3	198.8	23.66	754.0

### The identification of CMAs

The results of the CMA identification experiments are shown in Table 3. Though process conditions were fixed, the experimental results were quite different, indicating that the material attributes significantly affected the performance of *Astragali radix* ethanol precipitation process.

**Table 3:** CMA identification results.

Experimental no	Concentrates	Purity of flavonoids and saponins in the supernatant (μg/g dry mater)					Dry matter removal (Y_6_)
		Astragaloside IV (Y_1_)	Astragaloside II (Y_2_)	CG (Y_3_)	PG (Y_4_)	IFG (Y_5_)	
1	N1	1997	2294	2198	1408	1047	0.594
2	N2	1406	1024	2116	1161	973.0	0.464
3	N3	2985	2905	2501	1722	1527	0.471
4	N4	1366	1519	2468	1266	951.5	0.566
5	N5	563.4	661.2	1972	501.2	372.6	0.702
6	N6	415.2	420.0	2145	451.3	420.7	0.436
7	N7	749.2	722.0	1350	420.9	573.4	0.616
8	N8	437.2	457.8	1754	354.4	305.0	0.422
9	N9	614.0	781.3	2324	579.7	472.4	0.619
10	N10	424.6	239.4	1967	427.1	302.9	0.343

The correlation analysis of material attributes was carried out to find attributes with similar trends^37^. The Pearson coefficients are shown in Table 4. The Pearson coefficients among Astragaloside IV content (Z_1_), Astragaloside II content (Z_2_), PG content (Z_4_), IFG content (Z_5_) and electrical conductivity (Z_8_) was higher than 0.90. It means that one of them can roughly represent other three material attributes because they contained similar information. Electrical conductivity (Z_8_) was selected as the potential CMAs in the four material attributes because it is easy to measure. Other potential CMAs are CG (Z_3_), d-fructose content (Z_6_), and sucrose content (Z_7_).

**Table 4:** Pearson correlation coefficient of materials attributes and *P* value of significance test.

	Z_1_	Z_2_	Z_3_	Z_4_	Z_5_	Z_6_	Z_7_
Z_2_	0.948 (0.000)						
Z_3_	0.454 (0.188)	0.359 (0.308)					
Z_4_	0.947 (0.000)	0.906 (0.000)	0.660 (0.038)				
Z_5_	0.941 (0.000)	0.867 (0.001)	0.530 (0.115)	0.947 (0.000)			
Z_6_	− 0.243 (0.499)	− 0.371 (0.291)	0.099 (0.786)	− 0.077 (0.833)	− 0.062 (0.866)		
Z_7_	− 0.665 (0.036)	− 0.583 (0.077)	− 0.287 (0.421)	− 0.705 (0.023)	− 0.761 (0.011)	− 0.247 (0.492)	
Z_8_	0.943 (0.000)	0.960 (0.000)	0.547 (0.102)	0.953 (0.000)	0.907 (0.000)	− 0.241 (0.502)	− 0.585 (0.075)

Stepwise regression method was used to determine CMAs^38^. In this method, the term left in linear equations after stepwise regression indicates a CMA^38^. The ANOVA results of multiple linear regression analysis of each CQA using Eq. (3) are shown in Table 5. The determination coefficient (R^2^) of each model was higher than 0.70, indicating that the models can explain most of the variation of experimental data. However, these potential CMAs have no significant effect on the dry matter removal. It means that the determined material attributes were not main factors that influencing dry matter removal. According to the terms left in models, the CG content (Z_3_), the sucrose content (Z_7_), and the electrical conductivity (Z_8_) were found to be CMAs.

**Table 5:** Regression coefficient values, determination coefficients and ANOVA results.

Process parameters	Y_1_		Y_2_		Y_3_		Y_4_		Y_5_	
	Coefficient	*P* value	Coefficient	*P* value	Coefficient	*P* value	Coefficient	*P* value	Coefficient	*P* value
Constant	− 3401.126		− 3301.511		628.697		− 277.322		− 487.500	
Z_3_	− 0.8486	0.0381*	− 1.1012	0.0251*	1.1062	0.0021*			− 0.2446	0.0129*
Z_7_							− 1.8769	0.0148*	− 1.1572	0.0024*
Z_8_	3.1972	< 0.0001**	3.3332	< 0.0001**			1.3343	< 0.0001**	1.2904	< 0.0001**
R^2^	0.9482		0.9335		0.7126		0.9584		0.9915	

*P < 0.05.

**P < 0.01.

### The effects of CMAs and CPPs

**Figure 3:**
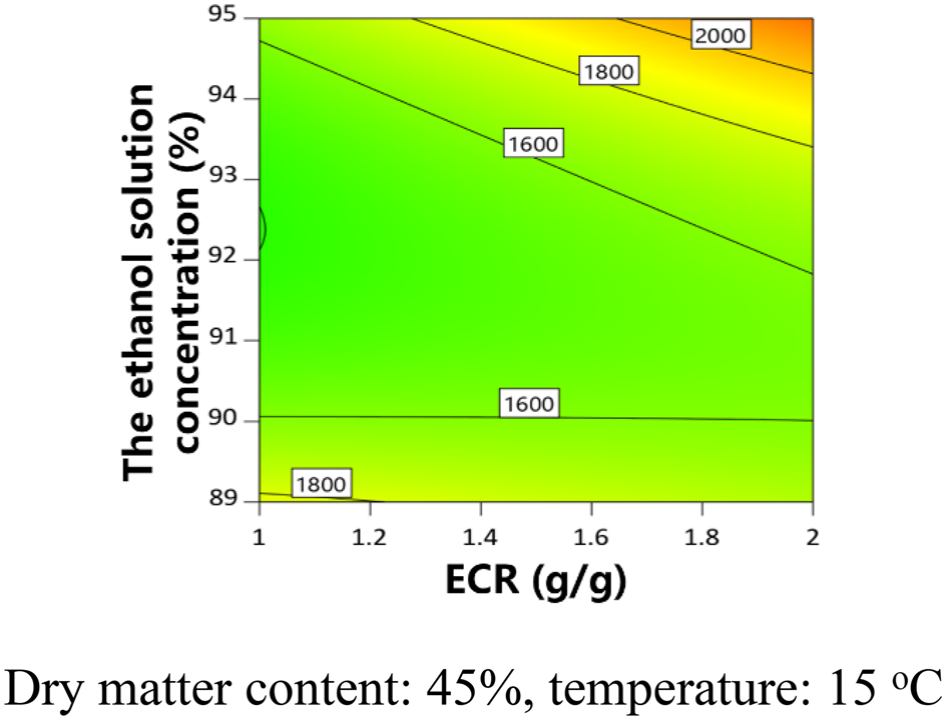
Contour plot of purity of Astragaloside II.

**Figure 4:**
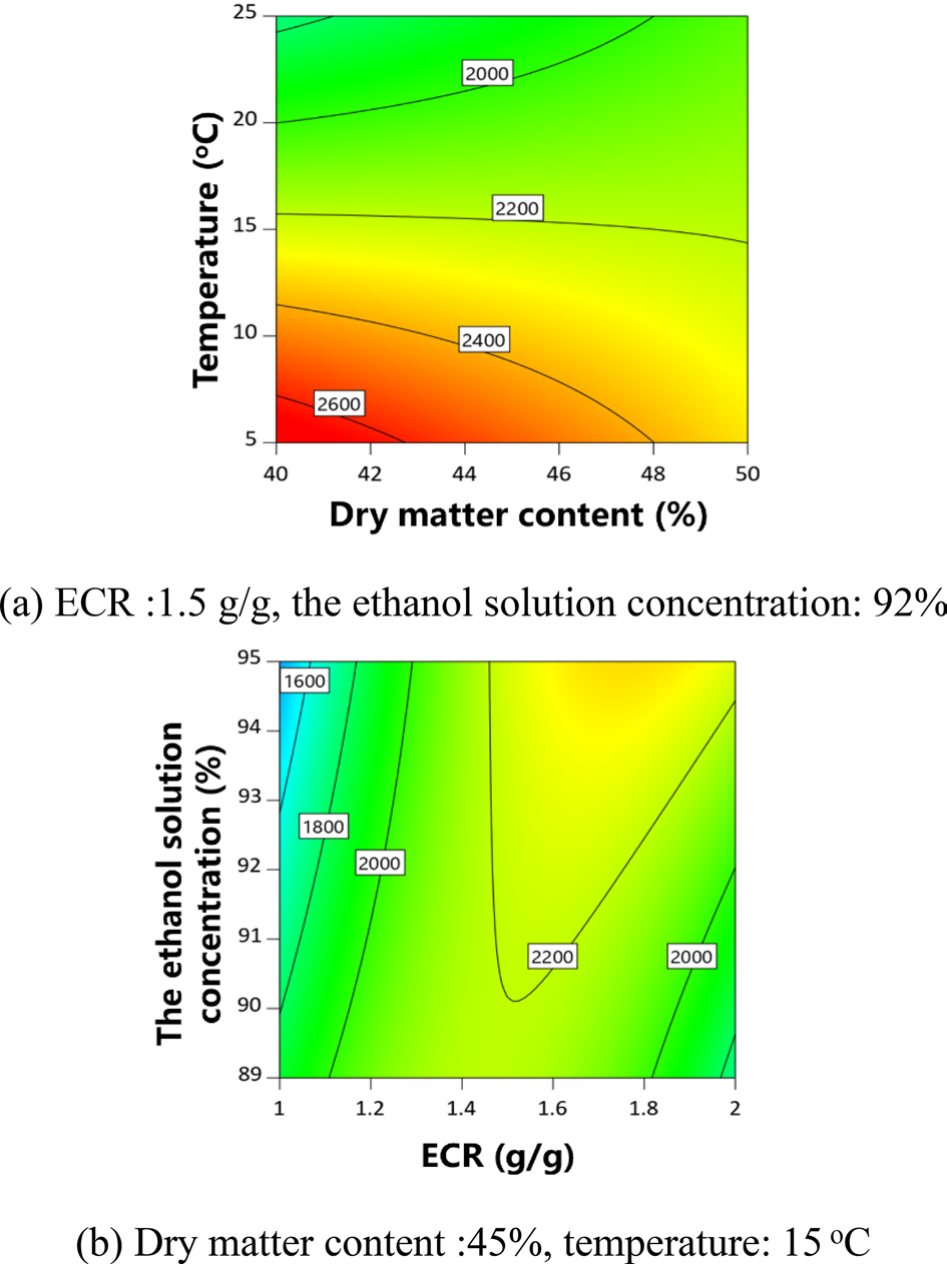
Contour plot of purity of CG.

**Figure 5:**
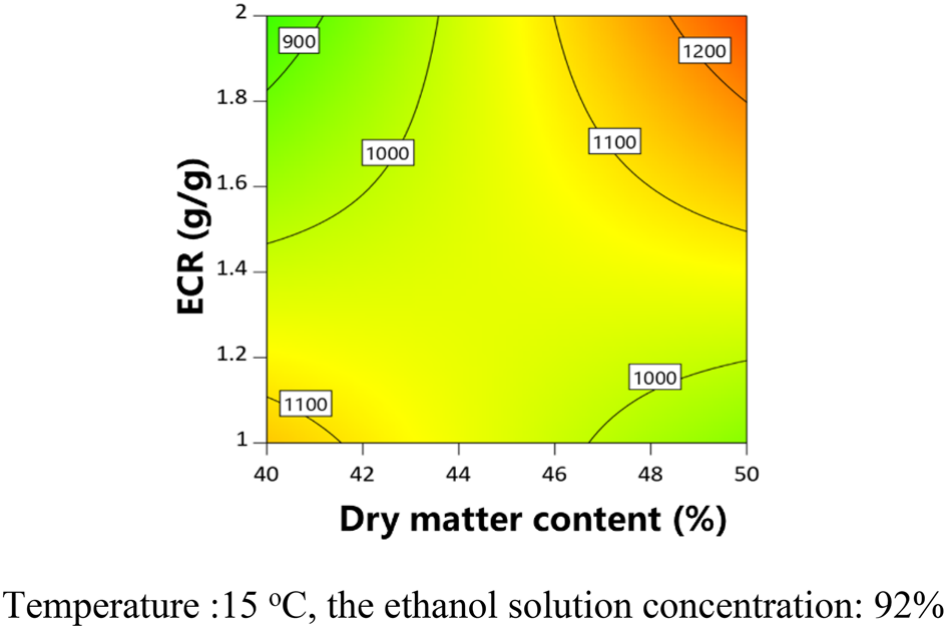
Contour plot of purity of IFG.

**Figure 6:**
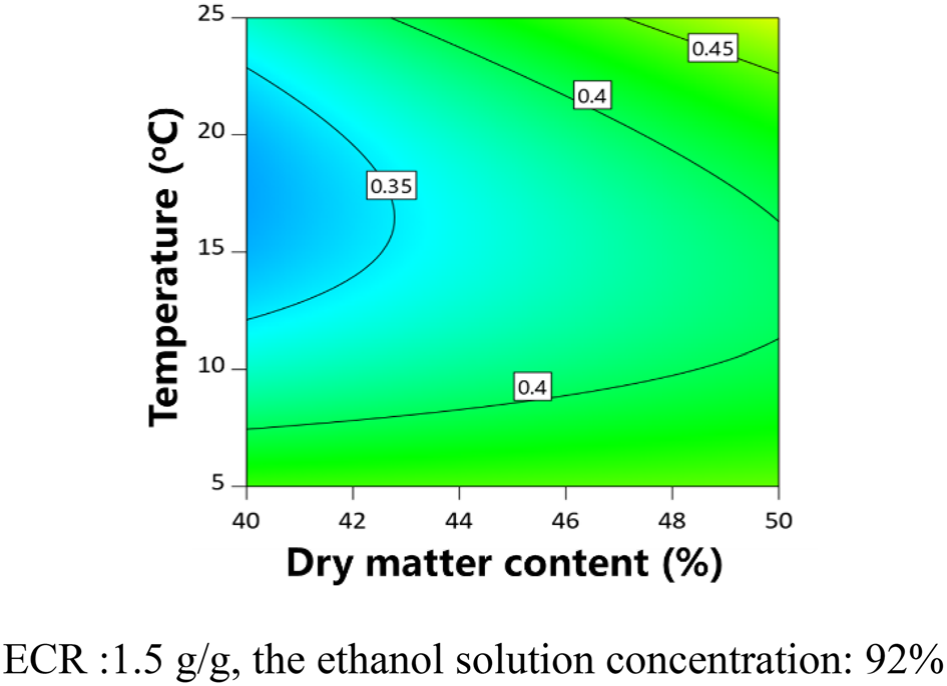
Contour plot of dry matter removal.

The partial regression coefficients and variance analysis results of the models are shown in Table 6. The *P* value of each model was less than 0.05, indicating that the model was significant. The adjusted determination coefficient (${\text{R}}_{{{\text{adj}}}}^{2}$) of each model was higher than 0.84. The contour plots were obtained to analyze the effects of CPPs on CQAs, as shown in Figs. 3, 4, 5, 6. The dry matter removal increased as dry matter contents increased. The purity of CG decreased as as temperature increased. The purity of Astragaloside IV was mainly affected by CMAs.The dry matter removal was mainly affected by CPPs. The purity of other flavonoids and saponins was affected by both CPPs and CMAs.

**Table 6:** ANOVA results for multiple regression models.

Process parameters	Y_1_		Y_2_		Y_3_		Y_4_		Y_5_		Y_6_	
	Coefficient	*P* value	Coefficient	*P* value	Coefficient	*P* value	Coefficient	*P* value	Coefficient	*P* value	Coefficient	*P* value
Constant	− 346.607		325,052.805		21,493.781		− 15,523.097		5054.318		− 0.7618	
X_1_					− 54.739	0.6925	683.552	0.6011	− 80.341	0.3744	− 0.0014	0.0170
X_2_			− 9928.885	0.0200	− 9026.701	0.0203	1888.456	0.7591	− 2716.876	0.7914	0.1024	0.0017
X_3_			− 7009.760	0.0804	− 221.201	0.6957					0.0131	0.0062
X_4_					− 181.541	0.0020	− 10.598	0.0174			− 0.0449	0.3841
Z_3_			− 0.614	0.0108								
Z_7_	− 2.816	0.0324					− 1.946	0.0147	− 3.419	0.0037		
Z_8_	1.846	0.0006	2.889	< 0.0001			0.691	0.0053	0.810	0.0025		
X_1_X_2_									60.954	0.0350		
X_1_X_4_					3.365	0.0834					0.0005	0.0532
X_2_X_3_			110.205	0.0045	152.158	0.0213						
X_1_^2^							− 7.643	0.0386				
X_2_^2^					1543.544	0.0278	− 622.660	0.0826				
X_3_^2^			37.338	0.0102								
X_4_^2^											0.0007	0.0128
R^2^	0.8703		0.9872		0.9431		0.9844		0.9449		0.9262	
${\text{R}}_{{{\text{adj}}}}^{2}$	0.8443		0.9744		0.8633		0.9625		0.9005		0.8524	
*P* value	< 0.0001		< 0.0001		0.0076		0.0003		0.0003		0.0036

### Design space development

A Monte Carlo method was performed using a self-coded MATLAB program (R2016a, Version 9.0, The Math Works Inc., USA) to calculate the design space based on the specific goals of process CQAs. The calculation process was introduced in previous work^36^. The acceptable ranges of the CQAs and the probability requirements for compliance are shown in Table 7. 1000 simulations were carried out to get the probability of every possible condition.

**Table 7:** The lower limits of process CQAs and probability requirements for compliance.

Process CQAs	Minimum	Acceptable probability of design space
Dry matter removal (%)	40	≥ 90%
Purity of Astragaloside IV (μg/g)	800	
Purity of Astragaloside II (μg/g)	700	
Purity of CG (μg/g)	1800	
Purity of PG (μg/g)	600	
Purity of IFG (μg/g)	600	

**Figure 7:**
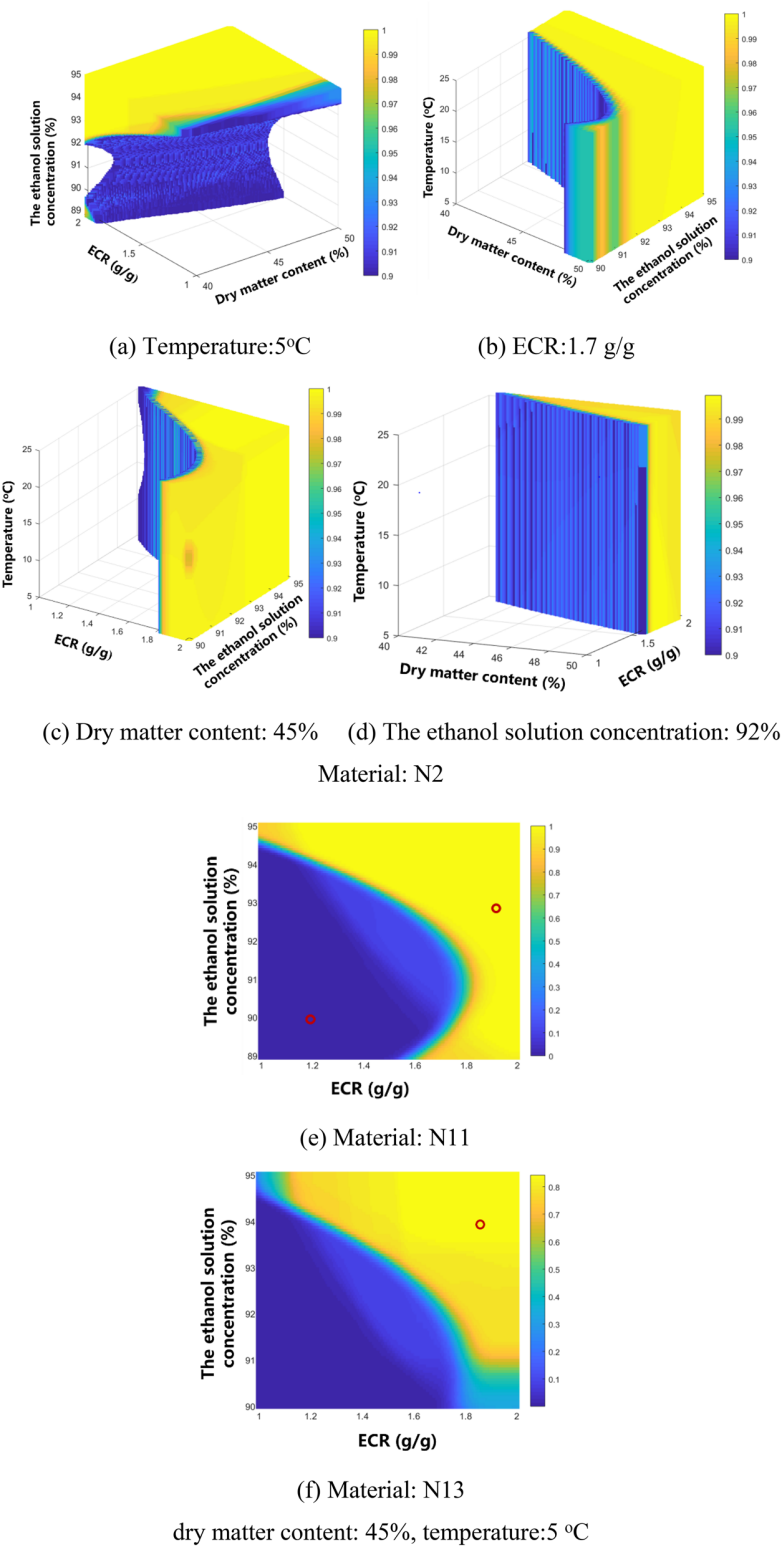
Design space and verification points (color bar refers to the probability of attaining the process CQA criteria; ○, verification points).

The conditions of design space were listed in Table S2, and shown in Fig. 7a–d. The design space was an irregular region.

### Control strategy of *Astragali radix* concentrates

In order to obtain a satisfactory supernatant, Inequalities (5) should be satisfied for CQA requirements listed in Table 7.

5 $$ \left\{ {\begin{array}{*{20}l} {b_{1}^{1} X_{1} + b_{2}^{1} X_{2} + b_{3}^{1} X_{3} + b_{4}^{1} X_{4} + b_{14}^{1} X_{1} X_{4} + b_{44}^{1} X_{4}^{2} + a_{0}^{1} \ge 0.4} \hfill \\ {c_{7}^{2} Z_{7} + c_{8}^{2} Z_{8} + a_{0}^{2} \ge 800} \hfill \\ {b_{1}^{3} X_{2} + b_{3}^{3} X_{3} + b_{23}^{3} X_{2} X_{3} + b_{33}^{3} X_{3}^{2} + c_{3}^{3} Z_{3} + c_{8}^{3} Z_{8} + a_{0}^{3} \ge 700} \hfill \\ {b_{1}^{4} X_{1} + b_{2}^{4} X_{2} + b_{3}^{4} X_{3} { + }b_{23}^{4} X_{2} X_{3} { + }b_{14}^{4} X_{1} X_{4} + b_{22}^{4} X_{2}^{2} + a_{0}^{4} \ge 1800} \hfill \\ {b_{1}^{5} X_{1} + b_{2}^{5} X_{2} + b_{4}^{5} X_{4} + b_{11}^{5} X_{1}^{2} + b_{22}^{5} X_{2}^{2} + c_{7}^{5} Z_{7} + c_{8}^{5} Z_{8} + a_{0}^{5} \ge 600} \hfill \\ {b_{1}^{6} X_{1} + b_{2}^{6} X_{2} + b_{12}^{6} X_{1} X_{2} + c_{7}^{6} Z_{7} + c_{8}^{6} Z_{8} + a_{0}^{6} \ge 600} \hfill \\ \end{array} } \right. $$

where superscripts refer to dry matter removal, purity of Astragaloside IV, purity of Astragaloside II, purity of CG, purity of PG, purity of IFG, and dry matter removal, respectively. The values of regression coefficients in Inequalities (5) can be found in Table 6. If the CMAs of a batch of *Astragali radix* concentrates meet Inequalities (5), the batch of *Astragali radix* concentrates is considered to be acceptable for ethanol precipitation. For a batch of acceptable *Astragali radix* concentrates, feasible process parameters can be chosen after calculation or selected from Table S2. A batch of *Astragali radix* concentrates is considered to be unacceptable when Inequalities (5) cannot be satisfied.

In industry, the process parameters are usually fixed. If the process parameters are fixed as follows: the ECR is 1.5 g/g, the dry matter content of concentrates is 45%, the ethanol solution concentration is 92% (v/v), and the temperature is 15 °C, Inequalities (5) can be simplified to Inequalities (6).

6 $$ \left\{ {\begin{array}{*{20}l} {Z_{8} \ge 1.52Z_{7} + 619.79} \hfill \\ {Z_{8} \ge - 0.21Z_{3} + 1376.52} \hfill \\ {Z_{8} \ge 4.22Z_{7} - 1100.10} \hfill \\ \end{array} } \right. $$

If a batch of *Astragali radix* concentrates with CMAs meeting Inequalities (6), this batch of *Astragali radix* concentrates is considered to be high quality material for the current parameter fixing process. If not, it is considered a low-quality material and should not be released for ethanol precipitation directly.

### Examples of material quality control and verification experiments

The CMAs of 3 batches of *Astragali radix* concentrates were measured and are shown in Table 8. According to Inequalities (6), *Astragali radix* concentrates of N12 were low quality *Astragali radix* concentrates, and it should not be released. The design space calculation results of N12 was show in Figure S3. The results shown that when the materials were unqualified, no matter how to change the CPP, the standards of CQA can not be achieved with a high probability.

**Table 8:** CMAs of *Astragali radix* concentrates for validation.

Concentrates no	Electrical conductivity (Z_8_, μS/cm)	CG contents (Z_3_, μg/g dry matter)	Sucrose contents (Z_7_, mg/g dry matter*)*	Quality grade
N11	1736	1292	617.6	High quality
N12	1568	1384	595.2	Low quality
N13	1631	1166	638.9	High quality

N11 and N13 were high quality *Astragali radix* concentrates. The verification experiment conditions and results are listed in Table 9 and Fig. 7e,f. All the predicted values were close to the experimental values, indicating that the models had good predictive performance.

**Table 9:** Verification conditions and results (*n* = 3).

CPPs and CQAs	V1	V2	V3
Concentrates	N11	N13	N11
Is it in the design space?	Yes	Yes	No
DM (%)	45	45	45
ECR (g/g)	1.9	1.9	1.2
The ethanol solution concentration (%)	93	94	90
Temperature (°C)	5	5	5
Dry matter removal (%)			
Experimental value*	49.23 ± 0.64	50.95 ± 2.46	37.88 ± 1.20
Predicted value	49.82	51.14	38.71
Purity of Astragaloside IV (μg/g)			
Experimental value*	1007.19 ± 60.07	899.73 ± 73.14	1083.41 ± 50.90
Predicted value	1119.77	865.77	1119.77
Purity of astragaloside II (μg/g)			
Experimental value*	948.76 ± 45.37	984.53 ± 28.71	893.08 ± 20.73
Predicted value	911.93	867.57	821.78
Purity of CG (μg/g)			
Experimental value*	2691.32 ± 133.66	2733.53 ± 97.48	2451.62 ± 24.05
Predicted value	2471.65	2539.55	2350.18
Purity of PG (μg/g)			
Experimental value*	1054.72 ± 46.97	765.27 ± 25.26	939.22 ± 25.75
Predicted value	1044.77	930.65	1074.02
Purity of IFG (μg/g)			
Experimental value*	887.45 ± 82.71	692.58 ± 23.22	869.80 ± 19.20
Predicted value	783.24	625.17	765.00

Experimental value*: average value ± SD.

## Discussions

### The effects of ethanol precipitation process on quality variation

The quality variations of most of Chinese medicines are from raw materials^39^. These variations may transmit from upstream intermediates to drug products. As a purification process, ethanol precipitation process is usually expected to reduce these variations.

The relative standard deviation (RSD) values of active component contents in the concentrates and ethanol precipitation supernatant of Experiment 1–10 were calculated, which are listed in Table 10. The RSD of active component contents were not decreased significantly. Active component contents in the precipitate of Experiment 2 was also analyzed. Astragaloside IV, Astragaloside II, CG, PG, and IFG were not found in the precipitate. It indicated that the variation of non-precipitated component contents could not be significantly reduced by ethanol precipitation process.

**Table 10:** RSD of flavonoids and saponins contents.

Materials	RSD of flavonoids and saponins contents				
	Astragaloside IV	Astragaloside II	CG	PG	IFG
Concentrates	0.782	0.731	0.201	0.598	0.632
Supernatants	0.778	0.795	0.166	0.610	0.589

For a Chinese medicine prepared with a series of unit operations containing ethanol precipitation, it is not enough to ensure the quality consistency of drugs only by the control of ethanol precipitation process parameters. Strict quality control of raw materials is sometimes more important. Mixing different batches of raw materials was an effective way to improve the quality consistency of raw materials. This strategy was introduced in many published works^39,40^.

### Selection of the cooling method

**Figure 8:**
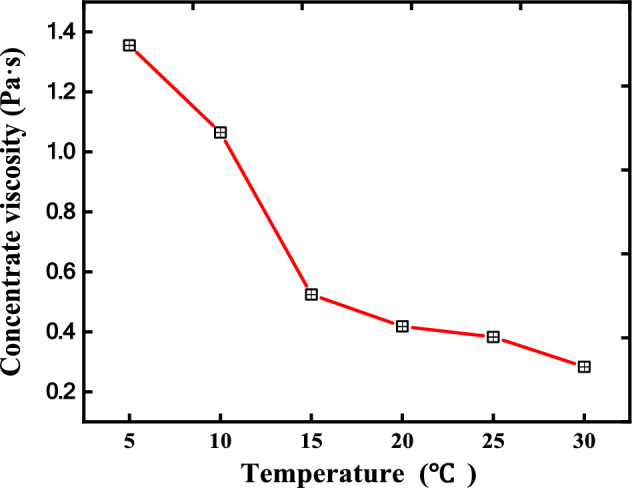
The effects of temperature on *Astragali radix* concentrate viscosity.

The viscosity of an *Astragali radix* concentrate at different temperatures was measured and shown in Fig. 8. *Astragali radix* concentrate viscosity increased about 1000 mPa·s when temperature lowered from 30 to 5 °C. For ethanol solution, viscosity increased about 0.8 mPa s when temperature lowered from 30 to 5°C^41^, which is much smaller than that of *Astragali radix* concentrate.

The increase of viscosity of an *Astragali radix* concentrate or an ethanol solution would lower the Reynolds number of continuous phase or dispersed phase^13^, respectively. Lower Reynolds numbers would result in worse mixing of two phases^13^. Because of larger viscosity increase of an *Astragali radix* concentrate, we did not try to cool a concentrate before mixing with an ethanol solution in this work. Because of smaller viscosity change after lowering temperature, the ethanol solution was cooled before mixing with a concentrate.

### Advantages and disadvantages

In industry, ethanol solutions and herbal concentrates were mixed in a stirred tank at most occasions. After that, the ethanol precipitation system was cooled by putting the stirred tank in a cold storage, or pumping cooling water through tank jacket. In general, the larger the volume of a stirred tank, the slower the temperature decreases. In this work, the ethanol solution was cooled before mixing with an *Astragali radix* concentrate. Therefore, the system temperature would be lowered after mixing. Compared with conventional methods in industry, the cooling time can be probably shortened.

The effects of two mixing methods of micromixing and stirring on ethanol precipitation were compared in previous work using *Codonopsis Radix* concentrates as the processing objects^13^. The mixing effect was better when using a membrane dispersion micromixer, which led to less loss of the active component^13^.

There are some shortcomings of this work. Firstly, there was no online detection of liquid temperature, neither a feedback control of cooling. In the future, if some automatic control method can be applied, such as Programmable Logic Controller (PLC) programs, the control accuracy of temperature can be further improved. Secondly, the models were established based on the results of a small number of experiments carried out in this study. In industry, it is necessary to accumulate production big data and update the models regularly to make the prediction results more reliable. This idea is also in line with the concept of “continuous improvement” mentioned in Dr. Yu’s paper^42^.

## Conclusion

In this study, a membrane dispersion continuous ethanol addition device which can achieve rapid cooling was developed for *Astragali radix* ethanol precipitation. The ethanol precipitation process was then optimized according to QbD concept. The experiments were carried out with different concentrates under fixed process conditions to identified the CMAs. CG content, the sucrose content, and the electrical conductivity were found to be CMAs. Definitive screening design was used to investigate the relationships among CPPs, CMAs, and CQAs. After model development, it is found that dry matter removal was mainly affected by CPPs. The purity of Astragaloside IV was mainly affected by CMAs. The purity of Astragaloside II, PG, and IFG were affected by both CPPs and CMAs. The design space was then calculated according to the probability of attaining process CQA standards. A material quality control strategy was proposed. High quality and low quality *Astragali radix* concentrates can be discriminated by the inequalities. Low quality *Astragali radix* concentrates should not be released for ethanol process directly. Verification experiments were carried out for high quality *Astragali radix* concentrates. The experimental results agreed well with the prediction results. The control strategy proposed in this work is promising to be used in other processes to improve batch-to-batch consistency of TCMs or herbal medicines.

## Supplementary information

Supplementary Information 1.

Supplementary Information 2.

## Data Availability

All data generated or analyzed during this study are included in this published article and its supplementary information files.

## Abbreviations

TCMs: Traditional Chinese medicines
QbD: Quality by design
CMAs: Critical material attributes
CQAs: Critical quality attributes
CPPs: Critical process parameters
CG: Calycosin-7-O-β-D-glucoside
PG: 9,10-Dimethoxypterocarpan-3-O-β-D-glucoside
IFG: 2-Hydroxy-3′,4′-dimethoxyisoflavane-7-O-β-D-glucoside
ECR: The mass ratio of ethanol solution to concentrate
AC: Active component contents
DM: Dry matter contents

## Superscripts

S: Supernatants
C: Concentrates
