# Supplementary material for: Optimization of membrane dispersion ethanol precipitation process with a set of temperature control improved equipment
Source: Sci Rep. 2020 Nov 4;10:19010. doi: 10.1038/s41598-020-75900-1 (PMC7643161; doi:10.1038/s41598-020-75900-1)
Supplement: Supplementary file 1 — Supplementary Information 1. [file 41598_2020_75900_MOESM1_ESM.docx]

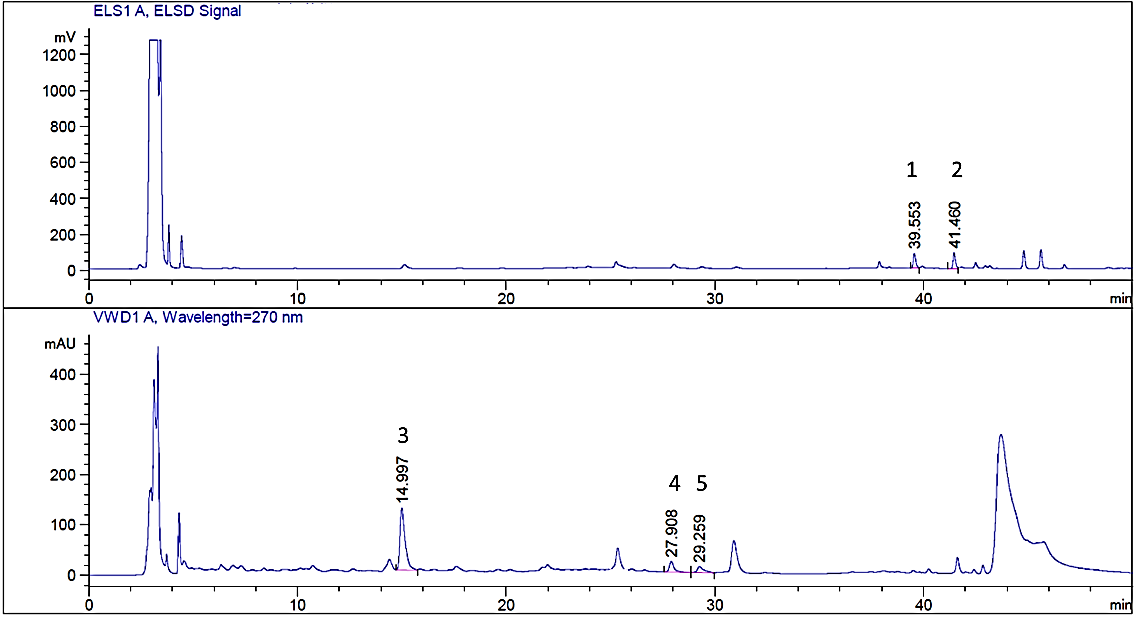


Figure S1. A typical HPLC-UV-ELSD chromatogram of an *Astragali radix* supernatant. Peak 1, 2, 3, 4, 5represented Astragaloside IV, Astragaloside Ⅱ, Calycosin-7-O-β-D-glucoside, 9,10-dimethoxypterocarpan-3-O-β-D- glucoside, and 2-hydroxy-3',4'-dimethoxyisoflavane-7-O-β-D-glucoside, respectively.


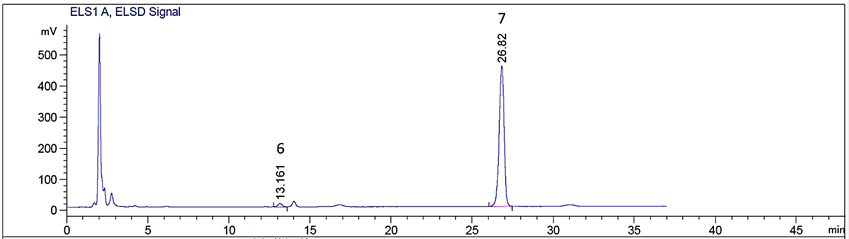


Figure S2. A typical HPLC-ELSD chromatogram of an *Astragali radix* concentrate. Peaks 6, 7 represented D-fructose, and sucrose, respectively.

Table S1 Temperature control in the experiments

| Temperature of concentrate (^o^C) | Temperature of refrigeration circulation device (^o^C) | Temperature of thermostatic bath (^o^C) | Temperature of mixture after stirring for 5min (^o^C) |
| --- | --- | --- | --- |
| 25$\pm$2 | -20$\pm$0.5 | 5$\pm$0.5 | 5$\pm$0.5 |
| 25$\pm$2 | -5$\pm$0.5 | 15$\pm$0.5 | 15$\pm$0.5 |
| 25$\pm$2 | 25$\pm$0.5 | 25$\pm$0.5 | 25$\pm$0.5 |


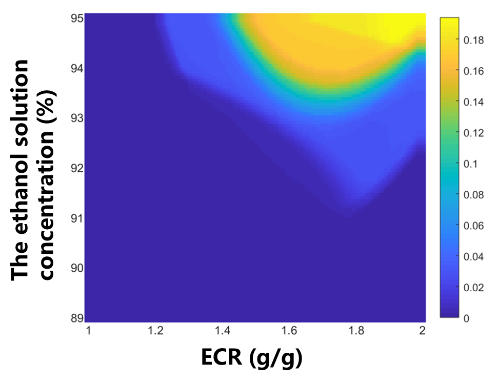


Figure S3. Design space calculation results of N12
